# Homotopic functional connectivity disruptions in glioma patients are associated with tumor malignancy and overall survival

**DOI:** 10.1093/noajnl/vdab176

**Published:** 2021-11-30

**Authors:** Andy G S Daniel, Carl D Hacker, John J Lee, Donna Dierker, Joseph B Humphries, Joshua S Shimony, Eric C Leuthardt

**Affiliations:** 1 Department of Biomedical Engineering, McKelvey School of Engineering, Washington University, St. Louis, MO 63130, USA; 2 Department of Neurological Surgery, Washington University School of Medicine, St. Louis, MO 63110, USA; 3 Mallinckrodt Institute of Radiology, Washington University School of Medicine, St. Louis, MO 63110, USA; 4 Department of Neuroscience, Washington University School of Medicine, St. Louis, MO 63110, USA; 5 Department of Mechanical Engineering and Materials Science, McKelvey School of Engineering, Washington University, St. Louis, MO 63130, USA; 6 Center for Innovation in Neuroscience and Technology, Washington University School of Medicine, St. Louis, MO 63110, USA; 7 Brain Laser Center, Washington University School of Medicine, St. Louis, MO 63110, USA

**Keywords:** functional connectivity, functional MRI, glioma, homotopic connectivity, resting state

## Abstract

**Background:**

Gliomas exhibit widespread bilateral functional connectivity (FC) alterations that may be associated with tumor grade. Limited studies have examined the connection-level mechanisms responsible for these effects. Given the typically strong FC observed between mirroring/homotopic brain regions in healthy subjects, we hypothesized that homotopic connectivity (HC) is altered in low-grade and high-grade glioma patients and the extent of disruption is associated with tumor grade and predictive of overall survival (OS) in a cohort of *de novo* high-grade glioma (World Health Organization [WHO] grade 4) patients.

**Methods:**

We used a mirrored FC-derived cortical parcellation to extract blood-oxygen-level-dependent (BOLD) signals and to quantify FC differences between homotopic pairs in normal-appearing brain in a retrospective cohort of glioma patients and healthy controls.

**Results:**

Fifty-nine glioma patients (WHO grade 2, *n* = 9; grade 4 = 50; mean age, 57.5 years) and 30 healthy subjects (mean age, 65.9 years) were analyzed. High-grade glioma patients showed lower HC compared with low-grade glioma patients and healthy controls across several cortical locations and resting-state networks. Connectivity disruptions were also strongly correlated with hemodynamic lags between homotopic regions. Finally, in high-grade glioma patients with known survival times (*n* = 42), HC in somatomotor and dorsal attention networks were significantly correlated with OS.

**Conclusions:**

These findings demonstrate an association between tumor grade and HC alterations that may underlie global FC changes and provide prognostic information.

**Key Points**

- Resting-state fMRI can identify functional connectivity differences between homologous brain regions in glioma patients.
- Homotopic connectivity is altered in glioma patients, dependent on tumor aggressiveness, and is associated with overall survival.

**Importance of the Study**

It has been shown that tumor heterogeneity and grade modify the extent of functional connectivity disturbances across the brain. High functional connectivity between homologous brain regions, or homotopic connectivity (HC) is a common feature of normal brain functioning. In glioma patients, the pathways facilitating these connections may be disturbed. Further, the degree of this disruption may depend on tumor aggressiveness. Thus, taken together, the variability of HC may have prognostic implications. Using resting-state fMRI, we assessed HC differences in low- and high-grade glioma patients and found associations between tumor severity and altered connections. Furthermore, HC was positively correlated with overall survival, with higher connectivity in the somatomotor network demonstrating a statistically significant difference in median survival. Therefore, HC may provide insight into glioma effects on global brain function.

The tumor burden on glioma patients extends beyond the proximal effects caused by the macroscopic lesion as tumor cells may be found throughout the cortex.^1,2^ In addition to distally seeded cells, given the highly connected nature of the brain, changes in the tumor microenvironment may elicit subsequent functional changes in seemingly healthy regions.^3,4^ These effects have been observed using resting-state functional MRI (rs-fMRI), which utilizes the fluctuations in blood-oxygen-level-dependent (BOLD) signals to identify brain regions that are temporally correlated.^3,4^ This process, termed functional connectivity (FC), can reveal the network similarity of spatially noncontiguous areas, enabling their study in healthy and diseased brains.^5–9^ Few studies have focused on the global cortical impact of glioma using FC.^3,4,10,11^ However, recent studies have demonstrated bilateral FC disruptions in glioma patients. One study demonstrated whole-brain FC alterations related to underlying tumor biology and cognitive impairments, with higher grade glioma resulting in greater deficits.^3^ Another study suggested that FC changes in resting-state networks are due to localized within-network damage, but this remains poorly understood.^4^

Defining alterations in interhemispheric connectivity may provide critical insights into the underlying mechanisms governing FC disruptions in glioma patients. For example, postmortem tract-tracing and diffusion-weighted MRI studies have shown a large proportion of callosal fibers interconnecting homotopic cortical regions.^12,13^ Furthermore, EEG and rs-fMRI studies have revealed bilateral functional relationships with high temporal synchrony between homotopic areas.^5,14,15^ Although such activity varied in strength by cortical area (e.g., primary vs association areas), homotopic connectivity (HC) is remarkably consistent across healthy subjects and is altered with the surgical disconnection of the corpus callosum and cortical lesions.^16–18^ However, few studies have investigated the impact of gliomas on homotopic connections.^19^ Glioma-induced homotopic disruption is supported by bilateral FC changes seen in unilateral glioma.^3^ Specifically, because increasing tumor grade has been associated with greater whole-brain FC disruptions, impaired HC in glioma patients is also likely to be dependent on tumor biology.^3^

Here, we investigated the relationship between glioma grade and HC. We hypothesized that glioma patients have reduced HC compared with healthy controls and that high-grade gliomas would be more impacted than low-grade gliomas. As other studies have shown that brain lesions may induce hemodynamic lags that disrupt FC,^8,20,21^ we decided to investigate whether homotopic lags would impact HC. Additionally, as high HC is a common feature of normal brain function, we hypothesized that its impairment is associated with overall survival (OS).^16,22,23^ Taken together, this work highlights the relevance of HC in understanding the global nature of brain dysfunction in glioma patients.

## Materials and Methods

### Subjects

Patients were retrospectively recruited from the neurosurgery brain tumor service, initially as part of a National Institutes of Health (NIH)-funded tumor database grant (NIH 5R01NS066905). All aspects of the study were approved by the Washington University in St. Louis (WUSTL) Institutional Review Board and the clinical data were retrospectively reviewed. Fifty patients with unilateral primary high-grade glioblastoma multiforme (GBM) and nine patients with low-grade glioma (LGG) underwent evaluation prior to surgical resection. The following inclusion criteria were used: diagnosis of primary brain tumor; age more than 18 years; and clinical need for a magnetic resonance imaging (MRI) scan, including rs-fMRI as determined by the treating neurosurgeon. Exclusion criteria included prior surgical resection for brain tumor, large tumor mass effect about the midline, prior radiation or chemotherapy, inability to have an MRI scan, and patients referred from an outside institution without rs-fMRI.

For control analyses, cognitively normal adult data (*n* = 30 subjects) were obtained from ongoing studies at the Alzheimer’s Disease Research Center (ADRC) of Washington University in St. Louis.^24^ These participants had a global Clinical Dementia Rating of 0 within 1 year of MRI.^25,26^

### Image Acquisition and Preprocessing

All imaging data were acquired using a Siemens 3T Trio or Skyra MRI scanner. Structural imaging included T1-weighted (T1w) magnetization prepared rapid acquisition gradient echo (MPRAGE), T2-weighted (T2w) fast spin echo, fluid attenuated inversion recovery (FLAIR) imaging, and postcontrast T1w fast spin echo in three projections. The rs-fMRI was acquired using an echo planar imaging sequence (voxel size = 3 mm cubic; echo time (TE) = 27 milliseconds; repetition time (TR) = 2.2–2.9 s; field of view = 256 mm; flip angle = 90°) for a total of 320 frames. For the healthy control subjects, the rs-fMRI scans were collected using a gradient-echo echo planar imaging sequence (voxel size = 3 mm cubic; TE = 27 milliseconds; TR = 2.2 s; field of view = 256 mm; flip angle 90°). Two rs-fMRI runs were acquired per subject with 164 frames per run (6 min). Preprocessing techniques have been previously described^9,27^ (see Supplementary material).

### Tumor Segmentation

Gliomas were segmented semi-automatically from multimodal image acquisitions (T1w, postcontrast T1w, T2w, and FLAIR) using the software application ITK-SNAP^28^. For patients with nonenhancing tumors, the tumor was defined using the T2w/FLAIR hyperintense volume, while for enhancing tumors, the tumor volume was defined as the T1w contrast-enhancing and T2w/FLAIR hyperintense regions. Segmented tumor voxels were excluded from homotopic parcels when calculating HC except where specifically noted.

### Homotopic Connectivity in Normal-Appearing Brain

To perform group-level HC analyses across the cortex, the left hemisphere of the 200-parcel Schaefer atlas was arbitrarily selected with each parcel assigned to one of seven resting-state networks.^29^ The parcels were axially reflected to produce mirroring parcels in the right hemisphere resulting in 100 homotopic pairs for comparison. This is similar to the voxel-mirrored and parcel approaches used by prior studies.^16,17,22^ The voxelwise timeseries within each parcel were averaged to create parcel-specific timeseries. Using Pearson correlation, the parcel-specific timeseries were correlated to their homologous parcel in the opposite hemisphere and Fisher z-transformed. Lesioned voxels were not included in HC calculations and parcels retaining less than 20 unaffected voxels were removed. This was motivated to preserve information of parcels with mixed content similar to previous reports.^30,31^ To compare to healthy subjects, each control was replicated (1–2 times) and randomly assigned to a glioma patient and treated identically, i.e., voxels corresponding to tumor-affected areas in glioma patients were also removed in the controls for timeseries calculations.^31^ HC differences between controls and glioma patients were assessed by averaging the connectivity strength of all the retained pairs or subdividing the parcels into cortical locations^32^ or resting-state networks and averaging the parcels within each subcategory.

### Homotopic Connectivity Between Tumor and Contralesional Hemisphere

To assess homotopic connectivity between tumor-disrupted areas and mirrored parcels, or tumor connectivity (TC), parcels with at least 20 lesioned voxels were separately analyzed by correlating the averaged lesioned voxel timeseries for each parcel with the corresponding homotopic parcel and was Fisher z-transformed. TC was then averaged for each parcel affected.

### Hemodynamic Lag Analysis

Homotopic lag was measured as previously described^21^ for each brain voxel by cross-correlating its BOLD timeseries to the homotopic voxel timeseries in the opposite hemisphere and identifying the time shift at which the cross-correlation between the voxel pair was maximal over the range ±5TR. Parabolic interpolation was performed to improve the estimation of the timepoint.^33^ To assess lags between homotopic parcels, the absolute lag of all voxels within each parcel was calculated and averaged together.

### Statistical Analysis

Statistical tests were performed in MATLAB, GraphPad Prism, and R. Two-sample (unpaired) *t*-tests were used to compare HC between subject groups where applicable. Simple linear regressions were implemented to evaluate associations between tumor severity and HC or hemodynamic lag. For HC and OS associations, the Dubey and Armitage-Parmer procedure for multiple comparisons correction was implemented.^34^ The log-rank test was used to compare Kaplan–Meier survival curves. Cox regressions were employed to compare the effects of covariates on survival. A *P*-value of .05 denoted statistical significance.

### Data and Materials Availability

Tumor data will be available upon request to E.C.L.

## Results

### Subject Characteristics

**Figure 1.**
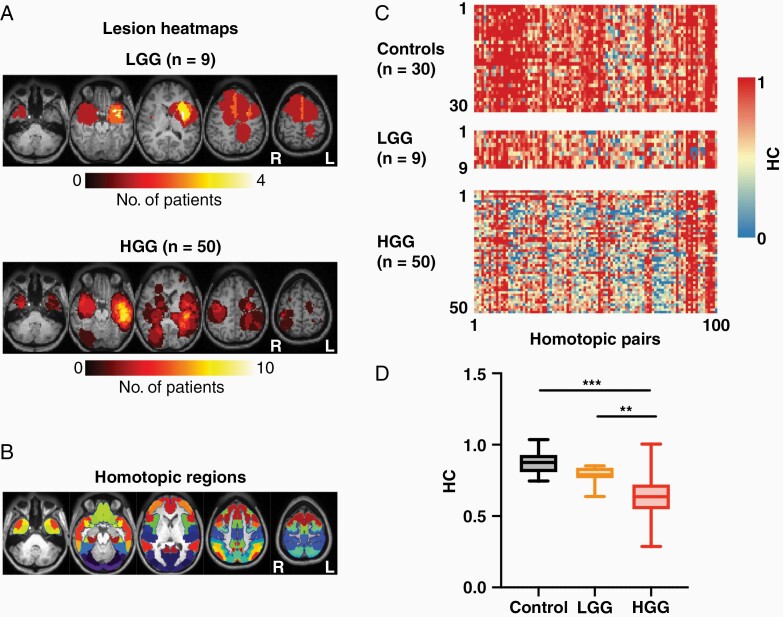
Assessing HC in low- and high-grade glioma patients. (A) Heatmaps showing LGG (top) and HGG (bottom) density in the 59-patient sample. (B) Homotopic pairs used for connectivity analyses. (C) Colormaps depicting FC of the 100 homotopic pairs in healthy (top), LGG (middle) and HGG (bottom) subjects. Warmer colors indicate stronger HC while cooler colors indicate weaker HC. (D) Global HC for each subject group obtained by averaging the tumor-free homotopic pairs in individual subjects. Controls were replicated to mimic the glioma tumor distributions with subsequent voxels removed before conducting connectivity analyses. HGG patients had significantly greater global HC than both LGG patients (*t* = 3.5, *P* = .002) and controls (*t* = 10.7, *P* < .0001). LGG patients were not significantly lower than controls (*t* = 2.2, *P* = .089). *P*-values were Bonferroni corrected for three tests.

Fifty-nine glioma patients (mean age; 57.5 ±14.5, 19 females) and 30 healthy controls (mean age; 65.9 ±10.8, 14 females) were retrospectively reviewed and included in this study (Table 1). Of the 59 patients, 9 were histologically diagnosed as low-grade (WHO grade 2) glioma and 50 were diagnosed as high-grade (WHO grade 4) glioma. Low-grade glioma (LGG) patients were diagnosed as oligodendrogliomas (*n* = 4) or astrocytoma (*n* = 5). All high-grade glioma (HGG) patients were diagnosed as glioblastoma multiforme (GBM). All LGG patients were IDH-1 mutated (*n* = 9) while all HGG patients were IDH wildtype (*n* = 50). Of the patients whose O^6^-methylguanine DNA-methyltransferase (MGMT) promoter methylation status was recorded, 17 out of 46 (37%) showed MGMT methylation. Figure 1A shows lesion distribution heatmaps to illustrate the tumor heterogeneity of LGGs and HGGs in the present patient sample. LGGs were more represented in the left hemisphere (7/9, 78.8%). For HGGs, there was a slight preference for tumors in the left hemisphere (30/50 patients, 60%). For patients with reported OS (all HGG, *n* = 42), mean OS was approximately 17.1 ±11.6 months. A template parcellation was used to extract HC between homotopic pairs for each patient (Figure 1C).

**Table 1.** Characteristics of Glioma and Control Subjects

Patient and control characteristics		
	Controls	All patients
Age, mean± SD (range)	65.9 ± 10.8 (53–89)	57.5 ± 14.5 (21–83)
Sex, *n*		
Female	14	19
Male	16	40
Tumor volume (cm^3^)	—	70.9 ± 53.4
Tumor histology, *n*		
Astrocytoma	—	5
Oligodendroglioma	—	4
Glioblastoma	—	50
WHO grade, *n*		
2	—	9
4	—	50
IDH mutation status, *n*		
Wildtype	—	50
Mutated	—	9
MGMT status, *n*		
Methylated	—	17
Nonmethylated	—	29
Missing	—	13
Extent of resection		
Gross total	—	23
Subtotal	—	20
Biopsy	—	7
Laser	—	9
OS^a^, mo, range	—	17.1 ± 11.6 (1.6–59.2)

^a^OS was known for only HGG patients (*n* = 42).

### HC Is Robustly Related to Glioma Severity

Visually, HC in normal-appearing brain appeared to be generally strong in controls, weaker in LGG patients, and even lower in HGG patients (Figure 1D). Quantitatively, this was demonstrated as HC in HGG patients, was significantly lower than both LGG (two-sample *t*-test, *t* = 3.49, *P* = .0021, Bonferroni corrected) and healthy controls (two-sample *t*-test, *t* = 10.67, *P* < .0001, Bonferroni corrected; Figure 1D). Although the mean of HC in LGG (mean = .79) was lower than that of controls (mean = .88), there was no significant difference between them (2-sample *t*-test, *t* = 2.2, *P* = .089, Bonferroni corrected).

**Figure 2.**
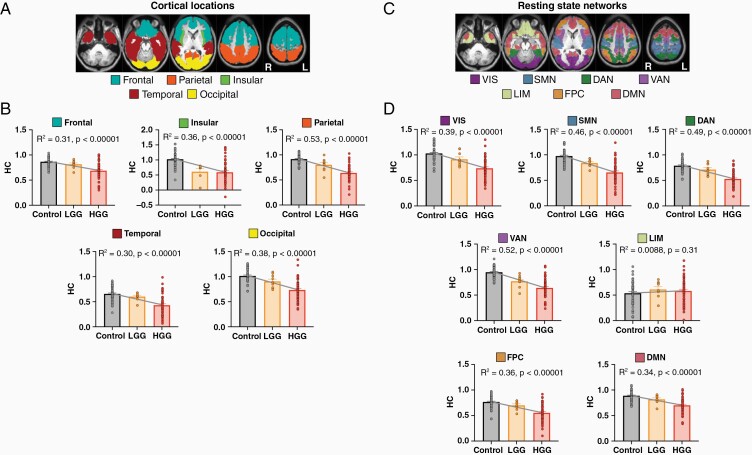
HC varies by cortical location and resting-state network but show robust associations with tumor grade. (A) Structural atlas template depicting cortex subdivided into frontal, parietal, insular, temporal, and occipital lobes. (B) HC in controls, LGG and HGG patients by cortical lobe. Across all cortical lobes there was a significant negative association between HC and clinical severity. (C) Resting-state network (RSN) parcellation used to identify HC. VIS: visual network; SMN: somatomotor network; DAN: dorsal attention network; VAN: ventral attention network; LIM: limbic network; FPC: fronto-parietal control network; DMN: default mode network. (D) HC in controls, LGG and HGG patients by RSN. Except for LIM, HC in all RSNs was significantly negatively associated with clinical severity.

The association between HC and tumor malignancy (Figure 1D) may be driven by parcels belonging to certain cortical locations or resting-state networks. Therefore, to investigate this possibility, we aggregated parcels by both cortical lobe (Figure 2A, B) and RSNs (Figure 2C, D). When grouped by cortical locations, the linear trend persisted for all regions with HGG patients consistently demonstrating lower HC on average compared to LGG patients and controls (Figure 2B). This effect was statistically significant for all cortical areas. The most variance was explained for the parietal lobes (*R*^2^ = 0.53, *P* < .00001) and the least explained variance was found for the temporal lobes (*R*^2^ = 0.30, *P* < .000001). When grouped by RSNs, a similar robust effect was observed in all networks except LIM (*R*^2^ = 0.0088, *P* = .31) (Figure 2D). For RSNs, the most variance was explained for the ventral attention network (VAN) areas (*R*^2^ = 0.52, *P* < .00001).

**Figure 3.**
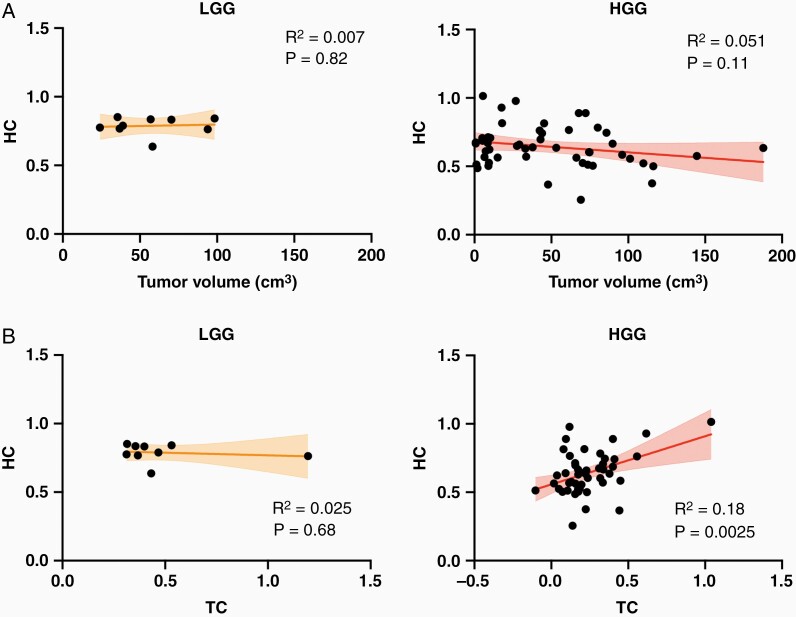
Investigating potential HC covariates. (A) Evaluating impact of tumor volume on HC in normal-appearing brain. In both LGG and HGG patients, there was not a statistically significant relationship between tumor volume and HC (LGG: *R* = 0.084, *P* = .82; HGG: *R* = −0.23, *P* = .11). (B) Determining impact of TC on HC. In LGG patients, TC was not associated with HC (*R* = −0.16, *P* = .68). However, there was a strong relationship between TC and HC in HGG patients (*R* = 0.43, *P* = .0025).

To further investigate the association between HC and glioma severity, we evaluated the potential effects of tumor size and TC. Figure 3A compares the effect of tumor size on HC in both LGG and HGG patients. In both cases, there was no significant correlation between tumor volume and HC although HC was weaker in larger tumors for HGG patients (LGG: *R* = 0.084, *P* = .82; HGG: *R* = −0.23, *P* = .11). In a subsequent analysis, we assessed the relationship between HC and TC. Interestingly, TC was only associated with HC for HGG patients (*R*^*2*^ = 0.18, *P* = .0025), but not for LGG patients (*R*^2^ = 0.025, *P* = .68). We also evaluated TC and HC for LGG and HGG patients normalized to lesion-matched controls (Supplementary Figure 1). HGG showed significantly lower HC and TC than LGG (two-sample *t*-test; LGG TC vs. HGG TC; *t* = 2.82, *P* = .0067, LGG HC vs. HGG HC; *t* = 3.23, *P* = .0021).

### Hemodynamic Lag Is Associated with HC in Glioma Patients

**Figure 4.**
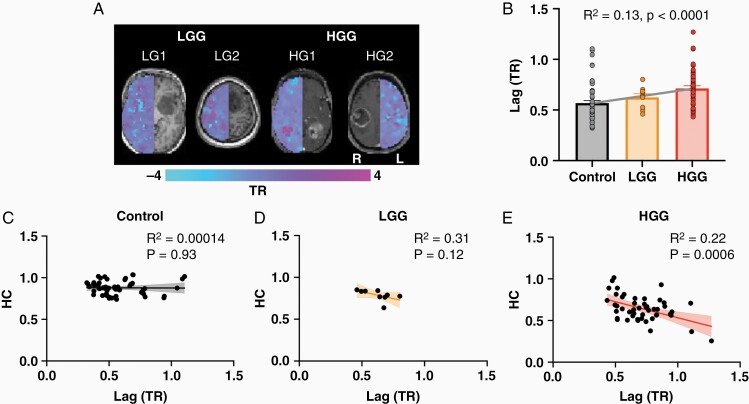
HC is correlated with homotopic lags in glioma patients. (A) Homotopic lag maps for two LGG and two HGG patients. Since lag calculations cross-correlate homotopic voxels, only the contralesional lags are displayed. The ipsilesional T1-weighted images show gliomas at corresponding axial slices. The time-shift scale ranges from −4 repetition time (TR) to +4 TR. (B) Mean absolute homotopic lag across the cortex is significantly associated with clinical severity (*P* < .0001). (C) HC is not correlated to homotopic lag in controls (*P* = .93). (D) HC is correlated with homotopic lag in LGG patients, but this was not statistically significant (*P* = .12). (E) HC was significantly correlated with homotopic lag in HGG patients (*P* = .0006).

The brain-wide relationship between HC and tumor severity suggests that a systemic driver may be involved. Therefore, lag analysis was performed to examine the potential impact of aberrant hemodynamics (Figure 4A). Figure 4A illustrates the contralesional lag maps of two LGG patients and two HGG patients. Regions of hemodynamic lagging and leading relative to the ipsilesional hemisphere can be observed. To quantify the global relationship between tumor grade and lag, we averaged the mean absolute lag of every parcel. Regression analysis found a strong association between hemodynamic lags and tumor grade with greater lags observed in HGG patients (*R*^2^ = 0.13, *P* < .0001; Figure 3B). HC was then correlated with lag across subject groups. No correlation was observed between HC and lags in controls (*R* = −0.012, *P* = .93). However, HC and lag showed a strong association in both LGG and HGG patients although this was only statistically significant for HGG patients (LGG: *R*^2^ = 0.31, *P* = .12; HGG: *R*^2^ = 0.22, *P* = .0006).

### Strength of HC Relates to Survival

Since tumor grade is strongly associated with patient outcomes and strong HC is a feature of normal brain function, we investigated whether HC could predict overall OS in our cohort of HGG patients (*n* = 42) at the time of their initial diagnosis. HC across all networks (mean RSN) was positively associated with OS, but this was not statistically significant (*R*^2^ = 0.092, *P*-adjusted = .071; Figure 4A). The contribution of HC in each RSN to OS was separately evaluated. SMN (*R*^2^ = 0.17, *P*-adjusted = .04) and DAN (*R*^2^ = 0.14, *P*-adjusted = .027) were significantly correlated with OS.

To further evaluate the relevance of SMN and DAN HC to OS, the patients were median split into low and high FC groups for each network, and survival was compared using Kaplan–Meier survival analysis (Figure 4B). This revealed a significant difference in survival for SMN (log-rank test, χ ^2^ = 4.7, *P* = .031) but not for DAN (log-rank test, χ ^2^ = 2.2, *P* = .13). Patients with low SMN HC had a median survival of 11.6 months compared with 17.1 months in the high SMN group. Univariate cox regression showed that SMN HC was a significant predictor of OS (Supplementary Table 1). After accounting for the potential influences of age and tumor size, SMN HC still maintained this effect (HR: 0.50, 95% CI: 0.25–0.97, *P* = .04). In a subset of HGG patients (*n* = 30) where MGMT methylation status was known and the extent of resection was limited to gross- and sub-total resections, the addition of these covariates on the multivariate model were investigated (Supplementary Table 2). In this model, only tumor volume (HR: 1.02, 95% CI: 1.01–1.04, *P* = .0007) and MGMT status (HR: 0.35, 95% CI: 0.13–0.97, *P* = .044) were statistically significant.

## Discussion

**Figure 5.**
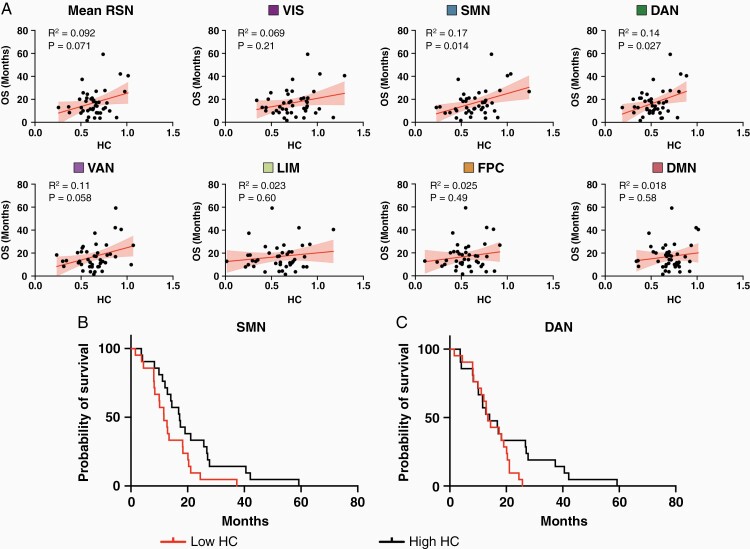
OS for HGG patients (*n* = 42) is associated with the HC of specific RSNs. (A) Plots depicting the association between RSN-specific HC and OS. Only SMN (*P* = .014) and DAN (*P* = .027) had statistically significant correlations with OS after multiple-comparisons correction. (B) Kaplan–Meier survival analysis for HGG patients comparing OS of patients with low SMN HC and patients with high SMN HC. Patients with high SMN HC had significantly longer median OS than patients with low SMN HC after accounting for tumor volume and age (HR: 0.50, 95% CI: 0.25–0.97, *P* = .04). (C) Kaplan–Meier survival analysis for HGG patients comparing OS of patients with low DAN HC and patients with high DAN HC. There was no OS difference between these two groups organized by DAN HC (log-rank test, χ ^2^ = 2.2, *P* = 0.13).

Whole-brain connectivity disruptions in glioma patients have been previously reported and demonstrate a remarkable association with tumor aggressiveness and cognitive impairments.^3^ Despite the potential clinical implications of these findings, the underlying functional connections responsible for these phenomena are not well understood. Therefore, we used the well-characterized concept of HC to investigate tumor-induced interhemispheric dysfunction in a sample of LGG and HGG patients.^16,22,35^ Several associations between HC and tumor severity were observed (Figure 1C, D, Figure 2). TC was only significantly associated with HC in HGG patients (Figure 3). Additionally, hemodynamic lags between homotopic areas were associated with tumor grade (Figure 4). Importantly, HC disruptions in HGG patients were also significantly associated with OS (Figure 5).

The highly symmetric nature of interhemispheric anatomical connections in the brain facilitates strong and stable functional connectivity between homologous areas.^12,23,36^ As a result, the growth of unilateral tumor lesions can lead to distorted connections that impact HC.^19,37^ The extent of disruption, however, is dependent upon several factors. Our findings suggest that tumor grade potentially plays an important role in the distortion of HC in glioma patients. It is not surprising that connectivity between tumor-altered cortical regions and their contralateral homologues would be severely weakened (Supplementary Figure 1). Gliomas can displace brain anatomy, including the astrocytic endfeet responsible for maintaining neurovascular coupling; an essential phenomenon for observing functional connectivity by fluctuating BOLD signals.^1,38,39^ Additionally, hypoxia in the microenvironment or glioma-secreted factors can induce neuronal cell death.^1,40^ However, it is intriguing that LGGs disrupt HC in these regions less than HGGs. Previous work has shown that functional areas can be found within tumor boundaries^9,41^ with greater frequency in LGGs compared to HGGs.^42^ Therefore, it is plausible that preserved function could result in less disrupted HC. The associative relationship between TC and HC seen only for HGGs also suggests a greater functional tumor burden. More invasive HGGs may disrupt local functional connections and seed glioma cells beyond the tumor mass more readily. Subsequently, these distal HGG cells may reduce global HC. This would support the findings of lower HC in HGGs across distinct locations and RSNs (Figure 2) as well as the greater amount of brain-wide functional alterations found in previous studies.^3,4^

Finding robust HC disruptions in glioma patients prompted us to consider altered neurovascular mechanisms given the hemodynamic nature of BOLD signals. Studies by Agarwal and colleagues previously identified decreased BOLD signal amplitudes in ipsilesional cortex despite no demonstrable deficits in patients across tumor grades.^43,44^ This finding suggests that normal neurovascular coupling should not be assumed in the vicinity of brain tumors and its impairment may contribute to our HC findings. However, we focused on extra-tumoral HC to minimize this contribution, proposing a more brain-wide phenomenon than previously reported. Hemodynamic delays due to cerebral lesions have been investigated primarily in stroke patients with limited glioma studies.^20,21,45,46^ As tumors grow, they disrupt bilateral neurovascular coupling and decrease synchronization of neural activity.^21,46^ This can explain homotopic lags reported in both animal and human studies.^21^ Thus, our finding of greater homotopic lags in more aggressive tumors provides complementary insights to previously reported results, suggesting that tumor grade may account for some differences. In LGGs and HGGs, lags explained 31% and 22% of the variances respectively, leaving open the possibility of unexamined neuronal contributions as shown in the work by Montgomery and colleagues.^46^

Strong synchronous activity between homologous regions is a mainstay of healthy brain functioning.^12,16,22,23^ Therefore, the degree of its impairment may act as a surrogate for global brain health. In fact, global HC was correlated with OS in HGG patients although this was not statistically significant. However, when restricting HC to specific brain networks, both SMN and DAN were significantly correlated with OS (Figure 5). To our knowledge, no one has previously reported a link between HC and OS in glioma patients. Although SMN HC produced statistically significant survival differences in univariate analyses, this was not observed in the multivariate analysis using age, tumor volume, MGMT status, and extent of resection as covariates (Supplementary Table 2). This suggests that SMN HC may not provide prognostic information beyond routinely collected clinical and genetic data. However, given the low patient sample used for this analysis (*n* = 30), the possibility of being statistically underpowered cannot be excluded. The consistency of these findings needs to be assessed in a larger prospective cohort of patients.

Further limitations may impact the results of this study. The lack of neurocognitive and behavioral data precluded further insight into the functional role of HC disruptions. Additionally, tumor location heterogeneity across glioma subtypes may have contributed to their HC differences. Predominantly frontal or insular tumors (LGG sample, Figure 1A) may elicit less HC disruptions in comparison to predominantly temporal lobe tumors (HGG sample, Figure 1A). Our study cohort was imbalanced with 9 LGG patients and 50 HGG patients. Future studies incorporating more LGG patients would help specify the influence of tumor location on global HC. Additionally, as all LGG patients were IDH-mutant and all HGG patients were IDH-wildtype, we were unable to test whether IDH-status conferred differences in HC beyond WHO grade status. HC, as an indirect measurement of brain activity, attempts to characterize tumor-associated effects beyond glioma-infiltrated cortex observable by structural MRI. HC is likely an indirect measurement of the state of the tumor microenvironment and glioma-neuronal interactions. Bidirectional interactions between neurons and gliomas involving neuronal hyperexcitability, direct neuron-glioma synapses, gap-junction interconnections, gliomas co-opting tumor-associated microglia and macrophages, and immunomodulatory mechanisms likely influence HC disruptions we observed.^47–49^ Hence, future studies combining global functional connectivity and tumor microenvironment assessments may advance mechanistic understanding of complex neuron-glioma interactions that could influence clinical decision-making.

In this study, we show that HC is altered in glioma patients and that this disruption may depend on tumor grade. Furthermore, the strength of HC in the somatomotor network is associated with OS. Thus, additional investigation of HC is needed in larger cohorts of low- and high-grade glioma patients to determine its clinical utility.

## Supplementary Material

vdab176_suppl_Supplementary_Materials

vdab176_suppl_Supplementary_Methods
