# Supplementary material for: Homotopic functional connectivity disruptions in glioma patients are associated with tumor malignancy and overall survival
Source: Neurooncol Adv. 2021 Nov 30;3(1):vdab176. doi: 10.1093/noajnl/vdab176 (PMC8694208; doi:10.1093/noajnl/vdab176)
Supplement: vdab176_suppl_Supplementary_Materials [file vdab176_suppl_supplementary_materials.docx]

**Supplemental Tables and Figure**

**Fig. S1.** Normalized connectivity of tumor-disrupted areas (TC) and normal-appearing brain (HC) in LGG and HGG patients. TC was markedly reduced (one-sample t-test; LGG: t = 3.3, *P* = 0.011; HGG: t = 17.46, *P* < 0.00001). HC was also significantly reduced for both LGG and HGG patients (one-sample t-test; LGG: t = 2.82, *P* = 0.023; HGG: t = 11.13, *P* < 0.00001). TC was less attenuated in LGG patients compared to HGG patients (two-sample t-test; LGG vs HGG: t = 2.82, *P* = 0.0067). There was a greater reduction of HC in HGG patients compared to LGG patients (LGG vs HGG: t = 3.23, *P* = 0.0021).


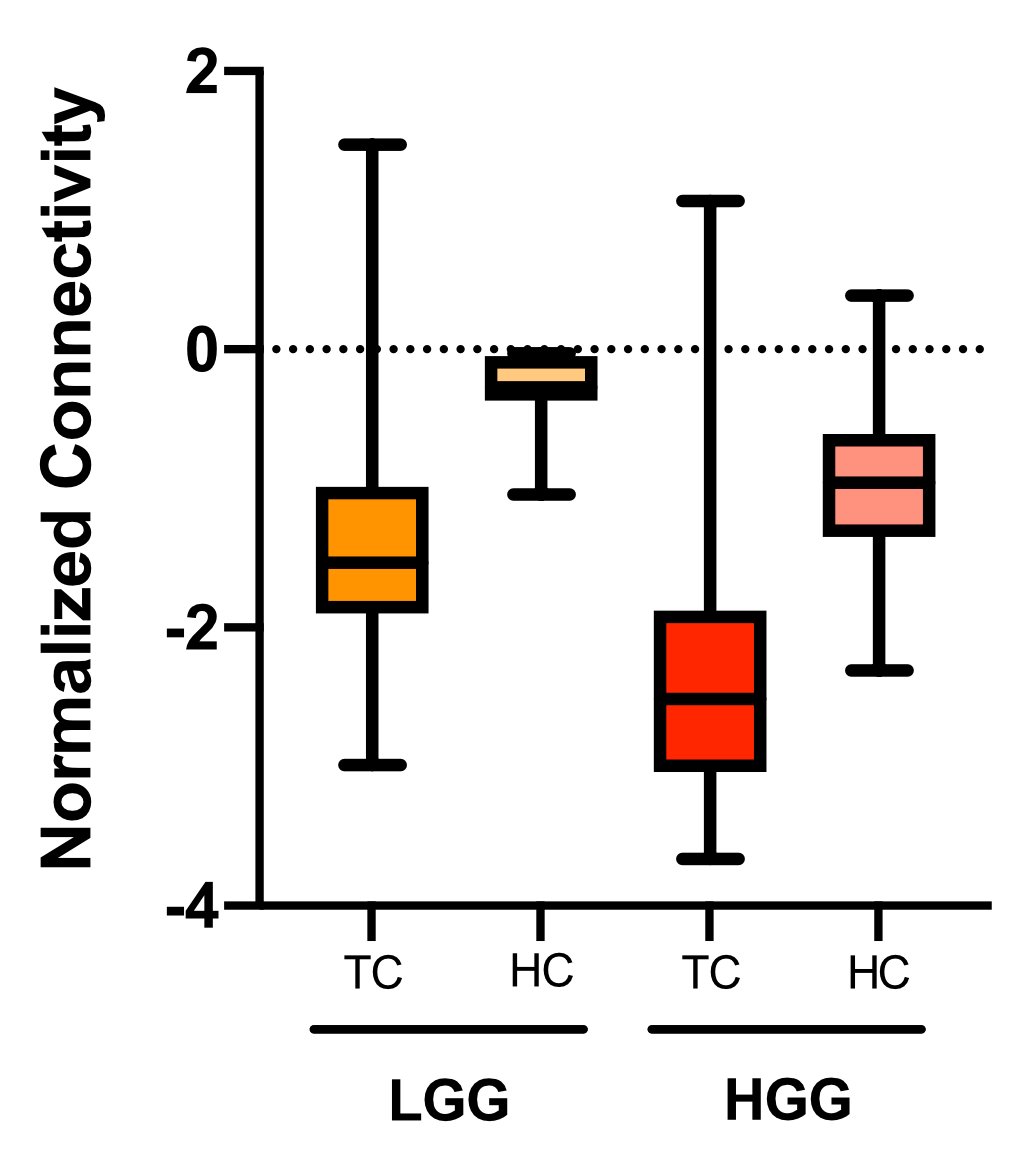


**Supplemental Tables**

**Table S1.** Univariate and multivariate survival analysis for HGG patients (n = 42)

| Characteristic | Univariate Cox | | Multivariate Cox | |
| --- | --- | --- | --- | --- |
|  | HR (95% CI) | *P*-value | HR (95% CI) | *P*-value |
| Age at initial Diagnosis | 1.01  (0.97,1.04) | 0.80 | 1.01  (0.97, 1.05) | 0.65 |
| Tumor volume (cm^3^) | 1.01 (1.00,1.02) | **0.0076** | 1.011  (1.00, 1.019) | **0.0085** |
| SMN HC = High | 0.50  (0.26, 0.95) | **0.034** | 0.50  (0.26, 0.97) | **0.04** |

**Table S2.** Univariate and multivariate survival analysis for HGG patients with MGMT status, gross-total, and subtotal resection (n = 30)

| Characteristic | Univariate Cox | | Multivariate Cox | |
| --- | --- | --- | --- | --- |
|  | HR (95% CI) | *P*-value | HR (95% CI) | *P*-value |
| Age at initial Diagnosis | 0.98  (0.94, 1.03) | 0.44 | 1.00  (0.94, 1.06) | 0.98 |
| Tumor volume (cm^3^) | 1.02  (1.01, 1.04) | **0.00007** | 1.02  (1.01, 1.04) | **0.0007** |
| SMN HC = High | 0.45  (0.21, 0.97) | **0.043** | 0.47  (0.18, 1.19) | 0.11 |
| MGMT = Methylated | 0.42  (0.17, 1.03) | 0.057 | 0.35  (0.13, 0.97) | **0.044** |
| Extent of resection = Gross total | 0.80  (0.38, 1.67) | 0.55 | 0.45  (0.19, 1.05) | 0.065 |
