# Supplementary material for: Homotopic functional connectivity disruptions in glioma patients are associated with tumor malignancy and overall survival
Source: Neurooncol Adv. 2021 Nov 30;3(1):vdab176. doi: 10.1093/noajnl/vdab176 (PMC8694208; doi:10.1093/noajnl/vdab176)
Supplement: vdab176_suppl_Supplementary_Methods [file vdab176_suppl_supplementary_methods.docx]

**Supplementary Methods**

*Preprocessing*

rs-fMRI preprocessing included correction for slice-dependent time shifts, removal of systemic odd-even slice intensity differences due to interleaved acquisition, and rigid body correction for interframe head movement. Atlas transformation was achieved by composition of affine transforms connecting the BOLD volumes with the T2-weighted and T1-weighted structural images, resulting in a volumetric time series resampled to 3-mm cubic voxels. BOLD time series were low-pass filtered to retain frequencies <0.1 Hz, spatially smoothed with a 6 mm full-width half-maximum Gaussian kernel, linear trends over each run were removed voxel-wise. Nuisance regressors included waveforms derived from head movement correction, white matter, cerebrospinal fluid, and the whole-brain (global) signal. Frame censoring was performed to minimize head motion effects. Frames in which the root mean square (evaluated over the whole brain) change in voxel signal intensity relative to the previous frame exceeded 0.5% (relative to the whole-brain mean) were excluded from the FC calculations.

*Normalized Connectivity*

TC and HC for both LGG and HGG patients were normalized to their lesion-matched controls to compare interhemispheric connectivity distributions of tumor-disrupted areas and normal appearing brain (Supplementary Fig 1.).
